# Supplementary material for: Machine Learning Accelerates Crystallization for Structure Determination
Source: Angew Chem Int Ed Engl. 2026 May 4;65(25):e1218503. doi: 10.1002/anie.1218503 (PMC13266958; doi:10.1002/anie.1218503)

checkCIF/PLATON report

Structure factors have been supplied for datablock(s) lcz017

THIS REPORT IS FOR GUIDANCE ONLY. IF USED AS PART OF A REVIEW PROCEDURE FOR PUBLICATION, IT SHOULD NOT REPLACE THE EXPERTISE OF AN EXPERIENCED CRYSTALLOGRAPHIC REFEREE.

No syntax errors found. CIF dictionary Interpreting this report

Datablock: lcz017

```
Bond precision:   C-C = 0.0169 A          Wavelength=1.54184

Cell:              a=13.2788(3)      b=13.2788(3)      c=15.8408(8)
                   alpha=90          beta=90           gamma=120
Temperature:       100 K
```

	Calculated	Reported
Volume	2418.94(16)	2418.94(16)
Space group	P 32	P 32
Hall group	P 32	P 32
Moiety formula	C24 H13 Ag3 F18 N6 O3	C15 H3 Ag3 F18 N6, C9 H10 O3
Sum formula	C24 H13 Ag3 F18 N6 O3	C24 H13 Ag3 F18 N6 O3
Mr	1099.02	1099.01
Dx,g cm-3	2.263	2.263
Z	3	3
Mu (mm-1)	15.835	15.835
F000	1578.0	1578.0
F000’	1585.77	
h,k,lmax	16,16,20	16,16,19
Nref	6904[3452]	4596
Tmin,Tmax	0.151,0.109	0.593,1.000
Tmin’	0.042	

```
Correction method= # Reported T Limits: Tmin=0.593 Tmax=1.000
AbsCorr = MULTI-SCAN
```

```
Data completeness= 1.33/0.67        Theta(max)= 78.134

R(reflections)= 0.0518( 4211)       wR2(reflections)= 0.1361( 4596)

S = 1.100                 Npar= 478
```

The following ALERTS were generated. Each ALERT has the format
test-name_ALERT_alert-type_alert-level.
Click on the hyperlinks for more details of the test.

Alert level B

```
PLAT915_ALERT_3_B No Flack x Check Done: Low Friedel Pair Coverage         37 %
```

Author Response: Due to insufficient data. The compound crystallizes in the chiral space group, but no chiral ligand is used, and the complex is no longer chiral.

Alert level C

```
PLAT042_ALERT_1_C Calc. and Reported MoietyFormula Strings  Differ     Please Check
              Calc: C24 H13 Ag3 F18 N6 O3
              Rep.: C15 H3 Ag3 F18 N6, C9 H10 O3
PLAT090_ALERT_3_C Poor Data / Parameter Ratio (Zmax > 18) ........       6.98 Note
PLAT250_ALERT_2_C Large U3/U1 Ratio for <U(i,j)> Tensor(Resd    1)        2.1 Note
PLAT342_ALERT_3_C Low Bond Precision on  C-C Bonds ...............    0.01685 Ang.
PLAT911_ALERT_3_C Missing FCF Refl Between Thmin & STh/L=    0.600          2 Report
                4 10  7,   1  1 18,
```

Alert level G

```
PLAT002_ALERT_2_G Number of Distance or Angle Restraints on AtSite         18 Note
PLAT003_ALERT_2_G Number of Uiso or U(i,j)  Restrained non-H-Atoms          3 Report
PLAT007_ALERT_5_G Number of Unrefined Donor-H Atoms ..............          1 Report
              H009
PLAT172_ALERT_4_G The CIF-Embedded .res File Contains DFIX Records          2 Report
PLAT186_ALERT_4_G The CIF-Embedded .res File Contains ISOR Records          1 Report
PLAT242_ALERT_2_G Low    'MainMol' Ueq as Compared to Neighbors of       C017 Check
PLAT242_ALERT_2_G Low    'MainMol' Ueq as Compared to Neighbors of       C01A Check
PLAT242_ALERT_2_G Low    'MainMol' Ueq as Compared to Neighbors of       C01B Check
PLAT242_ALERT_2_G Low    'MainMol' Ueq as Compared to Neighbors of       C01C Check
PLAT242_ALERT_2_G Low    'MainMol' Ueq as Compared to Neighbors of       C01F Check
PLAT720_ALERT_4_G Number of Unusual/Non-Standard Labels ..........         67 Note
              Ag01    Ag02    Ag03    F004    F005    F006    N007    F008
              O009    H009    F00A    O00B    F00C    F00D    N00E    F00F
              F00G    F00H    F00I    F00J    N00K    N00L    N00M    F00N
              C00O    H00O    F00P    F00Q    N00R    O00S    C00T    C00X
              C00Y    H00Y    C00U    C00W    H00W    C00V    H00V    C014
              C010    F00Z    C011    H011    C012    C013    H013    F015
              C016    C017    C018    H01A    H01B    H01C    C019    C01A
              C01B    C01C    C01D    F01E    C01F    C01G    C01H    H01D
```

```
              H01E    H01F    C01I
PLAT860_ALERT_3_G Number of Least-Squares Restraints ............         33 Note
PLAT883_ALERT_1_G Absent Datum for _atom_sites_solution_primary ..     Please Do !
PLAT912_ALERT_4_G Missing # of FCF Reflections Above STh/L=  0.600       111 Note
PLAT933_ALERT_2_G Number of HKL-OMIT Records in Embedded .res File         2 Note
               -1  2 19,   1  1 18,
PLAT941_ALERT_3_G Average HKL Measurement Multiplicity ..........        2.9 Low
PLAT969_ALERT_5_G The 'Henn et al.' R-Factor-gap value ..........      2.461 Note
              Predicted wR2: Based on SigI**2  5.53 or SHELX Weight 12.37
PLAT978_ALERT_2_G Number C-C Bonds with Positive Residual Density.         0 Info
```

---

0 **ALERT level A** = Most likely a serious problem - resolve or explain
1 **ALERT level B** = A potentially serious problem, consider carefully
5 **ALERT level C** = Check. Ensure it is not caused by an omission or oversight
18 **ALERT level G** = General information/check it is not something unexpected

2 ALERT type 1 CIF construction/syntax error, inconsistent or missing data
10 ALERT type 2 Indicator that the structure model may be wrong or deficient
6 ALERT type 3 Indicator that the structure quality may be low
4 ALERT type 4 Improvement, methodology, query or suggestion
2 ALERT type 5 Informative message, check

Validation response form

Please find below a validation response form (VRF) that can be filled in and pasted into your CIF.

```
# start Validation Reply Form
_vrf_PLAT042_lcz017
;
PROBLEM: Calc. and Reported MoietyFormula Strings  Differ     Please Check
RESPONSE: ...
;
_vrf_PLAT090_lcz017
;
PROBLEM: Poor Data / Parameter Ratio (Zmax > 18) ........       6.98 Note
RESPONSE: ...
;
_vrf_PLAT250_lcz017
;
PROBLEM: Large U3/U1 Ratio for <U(i,j)> Tensor(Resd    1)        2.1 Note
RESPONSE: ...
;
_vrf_PLAT342_lcz017
;
PROBLEM: Low Bond Precision on  C-C Bonds ..............    0.01685 Ang.
RESPONSE: ...
;
_vrf_PLAT911_lcz017
;
PROBLEM: Missing FCF Refl Between Thmin & STh/L=    0.600          2 Report
RESPONSE: ...
;
# end Validation Reply Form
```

It is advisable to attempt to resolve as many as possible of the alerts in all categories. Often the minor alerts point to easily fixed oversights, errors and omissions in your CIF or refinement strategy, so attention to these fine details can be worthwhile. It is up to the individual to critically assess their own results and, if necessary, seek expert advice.

PLATON version of 17/09/2025; check.def file version of 17/09/2025

---

duplicate check

No duplication found

Datablock lcz017 - ellipsoid plot

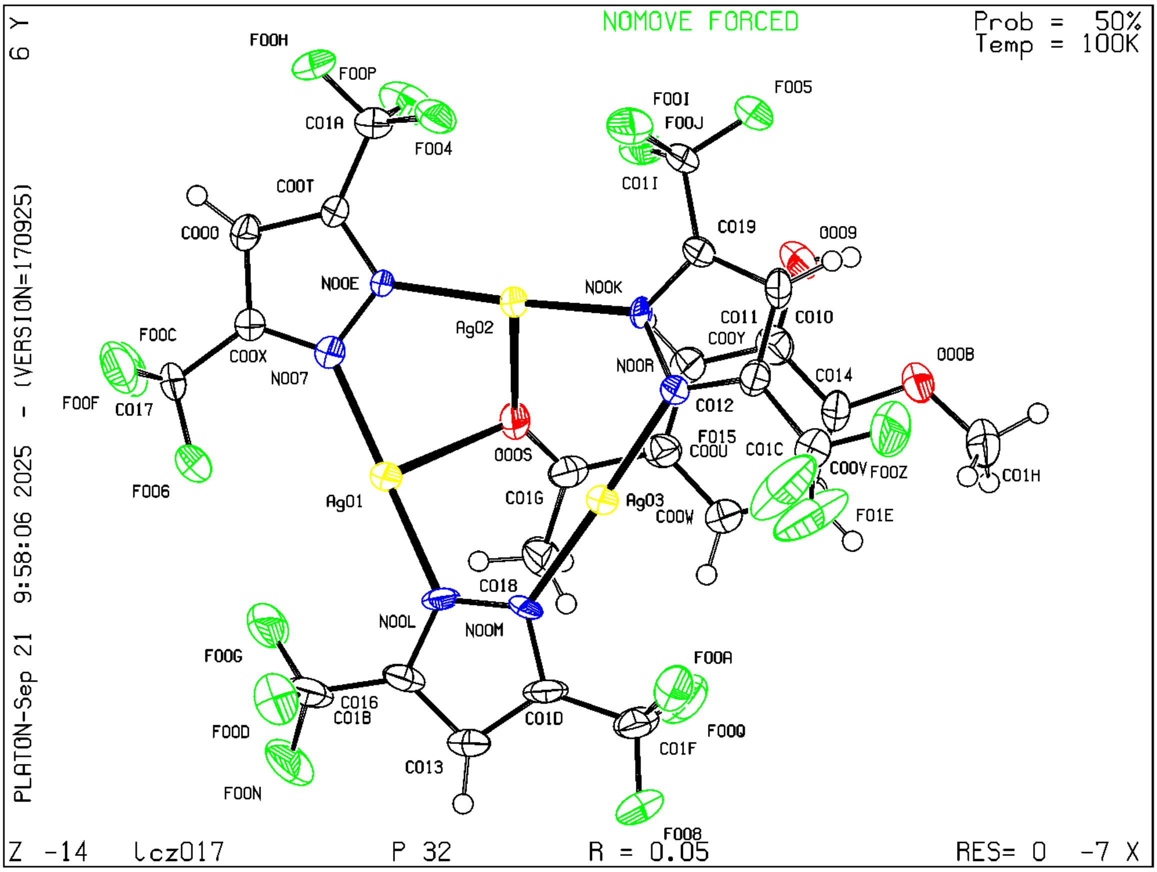

Supplement: Supplementary file 2 — Supporting File 2: anie72462‐sup‐0002‐DataFile.zip. [file ANIE-65-e1218503-s002.zip › supplementary_materials_crystallographic_data submit.zip!/cif_and_checkcif(1-114)/Ag3Pz3_17/Ag3Pz3_17_checkcif.pdf]
